# Assisted Reproductive Technologies (ART) in Latin America: The Latin American Registry, 2012

**DOI:** 10.5935/1518-0557.20140018

**Published:** 2014

**Authors:** Fernando Zegers-Hochschild, Juan Enrique Schwarze, Javier A. Crosby, Carolina Musri, Maria do Carmo B. de Souza

**Affiliations:** 1 Unit of Reproductive Medicine Clínica Las Condes, Chile; 2 Program of Ethics and Public Policies in Human Reproduction, University Diego Portales, Chile; 3 Clínica Monteblanco, Chile; 4 Fertipraxis - Human Reproduction - Rio de Janeiro - RJ, Brazil

**Keywords:** Assisted reproduction, biolaw, law

## Abstract

**Objective:**

This report examines information on Assisted Reproduction Technologies performed in Latin America (LA) during 2012.

**Methods:**

Multinational data were collected directly from 155 institutions in 14 countries. Individualized, case-bycase data include 47,326 ART cycles covering more than 80% of cycles performed in LA. Treatments included in vitro fertilization (IVF), intracytoplasmic sperm injection (ICSI), frozen embryo transfers (FET), oocyte donations (OD) and fertility preservation.

**Results:**

In 39% of ET IVF/ICSI was performed in women age 35-39 and 31% in women ≥40 years. Delivery rate (DR) per pick-up (OPU) in ICSI and IVF cycles, were 20.9% and 26.5%, respectively. Overall multiple births comprised 20.6% twins and 1.2% triplets. Furthermore, in OD, twins and triplets reached 27.8% and 2.4%, respectively. Pre term births in singletons were 14%. The relative risk of prematurity increased by 4.30 (95% CI 4.1-4.6) in twins, and 43.8 (95% CI 28.5-67.4) in ≥ triplets. Perinatal mortality increased from 25.2‰ in singletons, to 44.0‰ in twins and 80‰ in ≥ triplets. Elective single embryo transfer (eSET) was performed in only 1.4% of cycles with DR of 30% in women ≤34 years.

**Conclusion:**

Trends over the last 20 years show that eSET should be the way to go provided access is facilitated with public funding.

## INTRODUCTION

The Latin American Registry of Assisted Reproduction (RLA) was established in 1990, as the first multinational and regional registry collecting data on Assisted Reproduction Technologies (ART). For the first twenty years, summary data were obtained electronically via web page from every participating institution belonging to 12 countries in the region. Since 2010, new software has been developed and implemented, allowing for the collection of individualized case-by-case data from every treatment cycle. Data collection is, therefore, recorded individually starting from controlled ovarian stimulation (COS) until birth or miscarriage. Today, individualized data is obtained from ART treatments done in 155 institutions in 14 countries, covering more than 80% of ART cycles performed in the region. This report corresponds to the 24^th^ edition of RLA. Previous reports, from 1990 through 1998, are available as printed copies; from 1999 through 2009 as PDF files, which can be freely downloaded from the web page of the Latin American Network of Assisted Reproduction (REDLARA) at: http://www.redlara.com. Since 2010 onwards, the reports are published both in the JBRA Assisted Reproduction, the official journal of REDLARA, and online at http://www.redlara.com. This is the first report published simultaneously in Reproductive BioMedicine Online. The main objectives of RLA have been to disseminate information on ART procedures carried out in Latin America; monitor outcomes, as well as trends on safety and efficacy among centres and countries; empower infertile couples in their capacity to evaluate risks and benefits when requesting ART treatments; and develop a robust database for epidemiological studies. In this report, we are communicating information on availability, effectiveness, and perinatal outcomes of ART treatment performed during 2012 and babies born up to September 2013. It is also our aim to describe regional trends on how ART is practiced in the region, including the number of embryos transferred, multiple births and its impact on pre-term births and perinatal mortality.

## MATERIAL AND METHODS

### Data collection

One hundred and fifty five centres from fourteen countries (Supplementary Data) reported 47,326 ART procedures initiated between January and December 2012. Treatments include in vitro fertilization (IVF), intracytoplasmic sperm injection (ICSI), oocyte donation (OD) (both fresh and frozen), frozen embryo transfer (FET), and pre implantation genetic diagnosis and screening, registered together as PGD. As part of the accreditation programme, all participating institutions agree to have their data registered and published by the RLA. Given it is a multinational registry, no consent was required.

### Data validation

Information provided by each centre is checked by RLA central office for inconsistency before inclusion in the database. Any error or discrepancy not identified by the computer program is discussed with the centre, and the data is rectified if necessary.

### Limitations of data collection

Some centres lack complete description of events surrounding deliveries, such as weight of newborns, gestational age at delivery, perinatal outcome, or both. In fact, in 1530 deliveries (23%), no data were available on the weight of newborns. This lack of information, although small, is especially prevalent in assisted reproduction technology institutions that are not associated with obstetric units. Another potential limitation results from the fact that the inclusion of new cases, at the very start of a cycle, is not obligatory as in national registries, which are sometimes enforced by an independent body. Although we define an assisted reproduction technolgy cycle as initiated when ovarian stimulation is provided, in the RLA, new cases can be incorporated at the start of ovarian stimulation or at any time after that. Centres need to be certified by an independent body (accreditation programme) formed of a biologist and a clinician from a different country before their data can be included in the RLA. This is indeed a restriction for many centres that provide assisted reproduction technology treatments in the region. Although the reasons for not reporting are numerous, we estimate that a proportion of them refrain from reporting because their facilities would not pass the accreditation programme. Nevertheless, today, RLA has complete data from over 80% of procedures carried out in the region.

### Statistical analysis

Chi square test was used to analyse independence of categorical variables. When comparing two outcomes, the risk ratio (RR), and its corresponding 95% confidence interval (95% CI) are presented.

When multiple variable analyses were conducted (i.e. logistic regression or lineal regression), the dependent variables were considered significant if *P* < 0.05.

## RESULTS

### Participating centres

One hundred and fifty-five centres belonging to 14 countries reported their ART procedures carried out during 2012.

They included 31,857 initiated autologous fresh IVF/ICSI cycles; 10,073 frozen embryo transfers (FET) - both autologous and heterologous; 5,396 embryo transfers with donated oocytes (OD); and 1,764 initiated cycles for fertility preservation (FP).

The access to ART procedures, defined as the sum of IVF/ ICSI initiated cycles, FET and OD, per million women aged 15-45 years, reached 367 in 2012.

### Size of participating institutions

In 2012, excluding fertility preservation, a total of 47,326 cycles were reported.

The average number of initiated cycles reported was 309 (range 25-2552 cycles). Half of the centres reported less than 179 cycles, whereas six centres reported more than one thousand cycles.

The overall distribution of institutions according to the number of cycles reported is: 28% ≤ 100 cycles; 36% between 100 and 250 cycles; 18% between 251 and 500 cycles; 14% between 500 and 1,000 cycles; and only 4%, ≥ 1,000 cycles.

### ART procedure and access

Most cycles were reported by Brazil, representing 45% (*n*=21,452), followed by Argentina with 23% (*n*=11,031) and Mexico 12% (*n*=5,531) (table 1).

**Table 1:** Assisted Reproduction technology procedures and access in 2012

Country	Number of clinics	Assisted reproductive techniques							Access (** ** *)
		IVF/ICSI initiated cycles (*)	IVF (**)	ICSI (**)	FET(** *)	OD	FP(** **)	Total	
Argentina	25	6,461	504	5,515	3,027	1,543	429	11,031	1,193
Bolivia	1	215	148	62	14	8	923	237	96
Brazil	57	16,030	1,070	13,937	4,252	1,170	0	21,452	447
Chile	8	1,563	131	1,321	549	197	48	2,309	595
Colombia	11	977	293	622	262	247	13	1,486	139
Ecuador	6	608	216	324	165	154	107	927	254
Guatemala	1	100	38	62	7	17	0	124	37
Mexico	27	3,345	1,222	2,017	1,046	1,140	114	5,531	196
Nicaragua	1	91	46	41	0	9	0	100	67
Panama	1	245	7	192	86	33	9	364	452
Peru	6	1,264	298	875	430	547	114	2,241	308
Dominican R.	2	80	42	35	5	26	0	111	48
Uruguay	2	293	20	233	77	46	2	416	585
Venezuela	7	585	369	184	153	259	5	997	148
Total	155	31,857	4,404	25,420	10,073	5,396	1,764	47,326	367.0

(*) initiated cycles;

(**) oocyte pick ups;

(***) includes the transfer of own and donated oocytes;

(****) initiated fertility preservation cycles;

(*****) number of cycles/million of women 15-45 years

Out of 31,857 initiated autologous cycles, 1,008 were cancelled before aspiration (3.16%). Furthermore, in 1,025 follicular aspirations, no oocytes were recovered (3.22% of initiated cycles).

In 29,824 OPU, with at least one oocyte recovered, 4,404 (15%) corresponded to IVF and 25,420 (85%) to ICSI. Furthermore, 80.6% (*n*=24,047) of OPUs were followed by embryo transfer, and the main three reasons for no transfer were total embryo freeze (*n*=3,393), complete fertilization failure (*n*=991) and abnormal or no embryo development (*n*=823). Other reasons accounted for the remaining 570 cases.

One hundred and thirty six centres reported 10,073 FET cycles; and one hundred and thirty six centres reported 5,396 fresh OD cycles. Of these, 57% corresponded to exclusive donors, i.e. women undergoing controlled ovarian hyperstimulation (COS) and oocyte pick up with the only purpose of donating their oocytes; while 43% were egg-sharing, i.e. patients undergoing COS and oocyte pick-up, for an autologous treatment and simultaneously donated a proportion of their gametes to a third party.

### Pregnancies and deliveries

Tables 2 and 3 show the clinical pregnancy rate (CPR) and delivery rate (DR) per oocyte pick-up and embryo transfer. The CPR and DR per OPU in ICSI cycles, was 26.5% and 20.9%, respectively; while in IVF, it reached 32.8% and 26.5%, respectively (table 2). Thus, the relative risk to achieve a clinical pregnancy and a delivery for IVF compared with ICSI was 1.29 (95% CI 1.22 - 1.37) and 1.27 (95% CI 1.20 - 1.34) respectively.

**Table 2:** Clinical pregnancy rate and delivery rate IVF/ICSI (*) cycles in 2012.

ART procedure	Oocyte pick up (OPU)	Clinical pregnancy rate per OPU	Delivery rate per OPU
ICSI	25,420	26.5%	20.9%
IVF	4,404	32.8%	26.5%

(*) one case was labeled as “other”

**Table 3:** Clinical pregnancy rate and delivery rate OD, FET, FET(OD) cycles in 2012.

ART procedure	Embryo transfer (ET)	Clinical pregnancy rate per ET	Delivery rate per ET
OD	5,396	47.8%	39.9%
FET	7,880	31.6%	24.3%
OD (FET)	2,193	40.0%	28.1%

In OD cycles, the clinical pregnancy rate and delivery rate after fresh embryo transfers were 47.8% and 39.9%, respectively; which dropped to 40.0% and 28.1%, respectively, when FET were used. In FET with autologous oocytes, the CPR and DR were 31.6% and 24.3%, respectively (table 3).

### Age of women undergoing fresh autologous and heterologous IVF/ICSI and its effect on delivery rate

**Figure 1:**
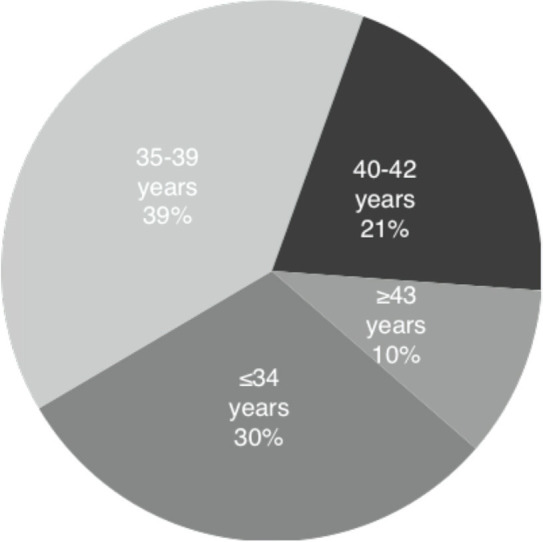
Age distribution of women undergoing autologous IVF/ICSI, 2012.

The mean age of women undergoing IVF/ICSI was 36 years (SD 4.6). The age distribution is shown in figure 1, whereby 30% of initiated cycles included women ≤34 years; 39% were women aged 35 through 39; 21% were women between 40 and 42 years; and 10% were women ≥43 years. Thus, 31% of women undergoing IVF/ICSI were ≥ 40 years.

**Figure 2:**
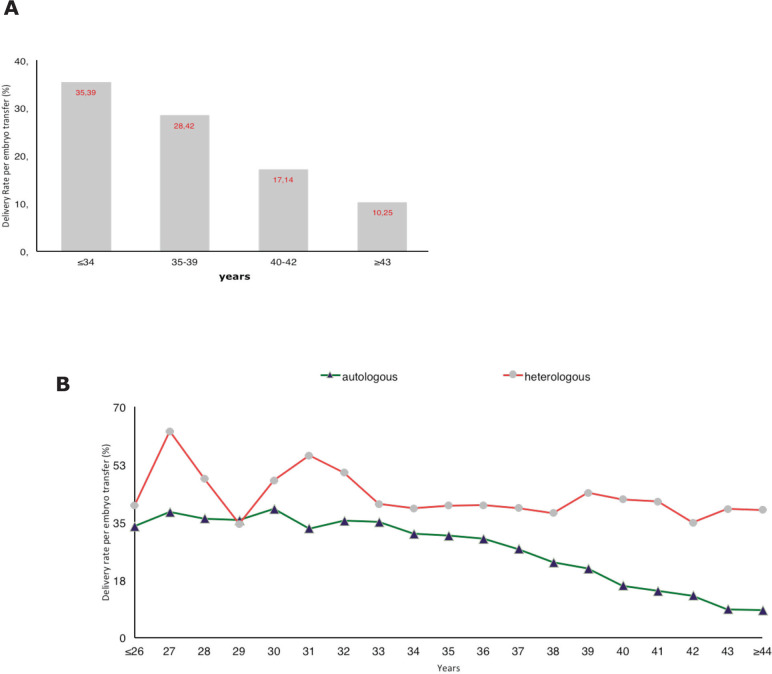
Delivery rate per embryo transfer according to age of the woman in autologous and heterologous IVF/ICSI, 2012 **(A)** Delivery rate per embryo transfer in different age categories of women in IVF/ICSI cycles, 2012; **(B)** devilery rate per embryo transfer according to age of woman in autologous and heterologous IVF/ICSI, 2012.

As expected, the delivery rate per embryo transfer in autologous reproduction significantly decreased from 35.4% in women ≤34 years to 10.3% in women ≥43 years *(P*<0.001) (Figure 2, panel A). On the other hand, when donor oocytes were used, the age of oocyte recipients did not systematically affect the outcome of embryo transfer. As seen in figure 2, panel B, DR/ET in oocyte recipients aged ≤34 years (*n*=421 ET) was 46.8%; 39.2% (*n*=943 embryo transfers) in women aged 35 through 39; 42.2% (*n*=1,288 embryo transfers) in women of 40 to 42 years; and 38.0% (*n*=2,744 embryo transfers) in women aged ≥43 years.

### Number of embryos transferred and multiple deliveries

#### Autologous reproduction

Table 4 shows the outcome of 24,047 IVF/ICSI transfers stratified by the number of embryos transferred. Overall, the mean number of embryos transferred was 2.2, and the proportion of 2 and ≥3 embryos were 55.7% and 28.8% respectively. The overall proportion of multiple births was 21.8%; of which 20.6% were twins and 1.2% triplet and higher. When 2 embryos were transferred, 21.5% of deliveries were twins and 0.4% triplets. The proportion of triplets increased to 3.3% when three embryos were transferred.

**Table 4:** Clinical pregnancy rate, delivery rate and gestational order according to the number of embryos transferred in fresh autologous IVF/ICSI cycles in 2012

Number of transferred embryos	Total ET		CPR/ET	Deliveries			
	Number	%		Total (number)	Singleton	Twin	≥Triplets
1	3,731	15.5%	16.9%	470	96.2%	3.8%	0.0%
2	13,392	55.7%	36.9%	4,025	78.1%	21.5%	0.4%
3	6,052	25.2%	37.8%	1,770	73.1%	23.5%	3.3%
≥4	872	3.6%	34.5%	216	82.4%	16.7%	0.9%
Total	24,047	100.0%	34.2%	6,481	78.2%	20.6%	1.2%

ET= embryo transfers

CPR= clinical pregnancy rate

#### Heterologous reproduction (OD)

Table 5 shows the outcome of 5,396 fresh OD transfers cycles stratified by the number of embryos transferred. The mean number of embryos transferred was 2.3, and the proportion of 2 and ≥3 embryos were 63.2% and 30.8% respectively The overall proportion of multiple births was 30.2 %; of which 27.8 % were twins, and 2.4 % triplets and higher. When 2 embryos were transferred, 27.8% of deliveries were twins and 0.9% triplets. The proportion of triplets increased to 5.6% when either three or four embryos were transferred (*P*< 0.001).

**Table 5:** Clinical pregnancy rate, delivery rate and gestational order according to the number of embryos transferred in fresh heterologous IVF/ICSI cycles in 2012.

Number of transferred embryos	Total ET		CPR/ET	Deliveries			
	Number	%		Total (number)	Singleton	Twin	≥Triplets
1	329	6.1%	35.6%	97	95.9%	4.1%	0.0%
2	3,408	63.2%	48.2%	1,376	71.2%	27.8%	0.9%
3	1,482	27.5%	49.1%	607	62.8%	31.6%	5.6%
≥4	177	3.3%	52.5%	72	66.7%	27.8%	5.6%
Total	5,396	100.0%	47.8%	2,152	69.8%	27.8%	2.4%

ET= embryo transfers

CPR=clinical pregnancy rate

#### Frozen/thawed embryo transfers (FET)

Table 6 shows 7,880 cases of autologous FET stratified by the number of embryos transferred. The mean number of embryos transferred was 2.1, and the proportion of 2 and ≥3 embryos were 59.7% and 22.8% respectively. The overall rate of multiple births was 20.9 %, of which 19.6 % were twins and 1.3 % triplets or more. When 2 embryos were transferred 20.7% of deliveries were twins and 0.2% triplets. The proportion of triplets increased to 4.8% when three embryos were transferred (*P*<0.001)

**Table 6:** Clinical pregnancy rate, delivery rate and gestational order according to the number of embryos transferred in FET cycles in 2012.

Number of transferred embryos	Total ET		CPR/ET	Deliveries			
	Number	%		Total (number)	Singleton	Twin	≥Triplets
1	1,381	17.5%	22.6%	227	97.4%	2.6%	0.0%
2	4,703	59.7%	33.9%	1,230	79.0%	20.7%	0.2%
3	1,617	20.5%	32.5%	417	69.3%	25.9%	4.8%
≥4	179	2.3%	32.4%	38	84.2%	13.2%	2.6%
Total	7,880	100.0%	31.6%	1,912	79.2%	19.6%	1.3%

ET= embryo transfers

CPR=clinical pregnancy rate

Table 7 shows 2.193 cases of FET with donated oocytes. The mean number of embryos transferred was 2.1. As expected, when compared with autologous transfers, the CPR and multiples was higher in each category of embryos transferred.

**Table 7:** Clinical pregnancy rate, delivery rate and gestational order according to the number of embryos transferred in FET(OD) cycles in 2012.

Number of transferred embryos	Total ET		CPR/ET	Deliveries			
	Number	%		Total (number)	Singleton	Twin	≥Triplets
1	290	13.2%	25.9%	56	96.6%	5.4%	0.0%
2	1,420	64.8%	38.5%	407	76.7%	32.1%	0.3%
3	453	20.7%	39.3%	144	63.2%	33.3%	3.5%
≥4	30	1.4%	36.7%	9	55.6%	33.3%	11.1%
Total	2,193	100.0%	37.0%	616	78.8%	24.0%	1.1%

ET= embryo transfers

CPR= clinical pregnancy rate

#### Elective singe and dual embryo transfer (eSET & eDET)

Elective single embryo transfer (eSET) and elective double embryo transfer (eDET) accounted for 1.4% (*n*=347) and 21.0% (*n*=5,038) of embryo transfers respectively.

The overall DR/ET was 24.5% with eSET and 38.8% with eDET. These values were significantly higher than non-elective SET and DET where DR were 11.4% in 3,384 transfers and 24.9% (*n*=8,354), respectively *P*<0.0001).

When stratified by the age of female partner, in women ≤34 years the DR after eSET and eDET increased to 30.0% and 42.0%, respectively (*P*<0.0001). Similarly, in OD fresh cycles the DR/ET after eSET and eDET were 29.5% (*n*= 329 ET), and 40.4% in eDET respectively (*n*=3,408 ET).

#### Perinatal outcome

The duration of gestation was reported in 13,313 deliveries, of which 10,048 were singletons, 3,054 twins, and 211 triplets or more. Among singletons, the mean gestational age at delivery was 37 weeks of amenorrhoea, 35 weeks of amenorrhoea in twin deliveries and 32 and 29 weeks in triplets and quadruplets, respectively (*P* < 0.001).

The percentage of preterm birth, among singletons was 14.0% (*n* = 1,405). The relative risk of preterm birth for twins increased by 4.30 (95% CI 4.1 to 4.6), and 43.8 (95% CI 28.5 to 67.4) for triplets and higher order multiples. Furthermore, the percentage of very preterm birth (i.e. before completing 32 weeks of amenorrhoea in singletons was 1.7% (*n* = 171), 7.1%% (*n* = 217) in twins and 32.2% (*n* = 68) in triplets and higher order multiples (*P* < 0.0001).

Table 8 shows perinatal mortality according to gestational order. Singletons had a perinatal mortality of 25.2 per thousand, compared with 44.4 per thousand in twins and 80.7 per thousands in triplets or higher order multiples (*P* < 0 .0001).

**Table 8:** Perinatal mortality according to gestational order in 2012

		Singleton			Twin			≥Triplets	
	LB	SB	ND	LB	SB	ND	LB	SB	ND
***IVF/ICSI***	4947	102	20	2571	68	29	213	11	12
***FET***	1,473	34	7	715	20	13	67	4	2
***OD***	1,460	35	7	1124	52	22	146	2	5
***FET (OD)***	451	6	4	282	10	4	18	3	0
***Total***	8,331	177	38	4,692	150	68	444	20	19
***Perinatal mortality per*** ***1000***		25.2			44.4			80.7	

LB= live borns; SB= still borns; ND= neonatal death

Thus, compared with singletons, the relative risk of perinatal mortality among twins was 1.4 (95% CI 1.3 to 15.), and 5.4 (95% CI 4.4 to 6.6) among triplets and higher order multiples.

#### Spontaneous abortion rate

In fresh autologous IVF and ICSI pregnancies, the overall spontaneous abortion rate was 18.4%. When stratified by age, spontaneous abortion rate increased from 14.4% in women aged 34 years or younger, to 18.5% in women aged 35-39 years; 24.1% in women between 40 and 42 years; and 20.4% in women over 42 years (*P* < 0.001). Within each age category, the rate of spontaneous abortion did not differ significantly when comparing women with and without PGD.

In fresh-oocyte recipients, the spontaneous abortion rate was 15.4%, and no significant differences were found when stratified by age of recipients. Furthermore, spontaneous abortion rate in pregnancies after autologous FET was 22.0%. No subgroup analysis was carried out in this case, as the RLA reports the age of women at the time of embryo transfer not at the time of embryo freezing.

#### Pre-implantation Genetic Diagnosis (PGD)

The RLA registers PGD and PGS together. Seventy-four centres from Argentina, Brazil, Chile, Colombia, Mexico, Panamá, Peru and Venezuela reported 1,664 cycles of PGD. Of these, 20 cases were polar body biopsies, 65 cases were biopsies of cleaving-embryos, whereas 1579 PGD were carried out in blastocysts.

Overall, 708 embryo transfer cycles were carried out. The mean age of women was 38 years (22-48 years). A mean of four embryos were analysed in each cycle, and a mean of one embryo was reported as normal. Out of 264 clinical pregnancies and 218 deliveries, a total of 252 babies were born; none of which was reported as having birth defects.

#### Assisted hatching (AH)

Institutions in Argentina, Brazil, Chile, Ecuador, Mexico, Peru, Venezuela and Uruguay reported 4,775 cycles with AH and 4,243 embryo transfers, generating 1,441 clinical pregnancies and 1,082 deliveries.

Of these, 846 were reported as singletons, 220 as twins and 16 as triplets. The mean age of the women undergoing assisted hatching was 38 years (18 to 55 years).

#### Intrauterine insemination (IUI)

The results of IUI cycles are presented in Table 9. These are reported by clinics located in nine different countries, either with semen of the husband (IUI-H) or donor (IUI-D). Eighty-three clinics of ten countries reported 5,372 cycles of IUI-H. The delivery rate per cycles was 12.3%. of which 9.3% were twin and 1.4% triplets and higher-order multiples. Sixty five clinics in 10 countries reported 1,029 cycles of IUI-D. The delivery rate per cycles was higher, 17.8%. The multiple-delivery rate was 12.0%: 8.2% twins and 3.8% triplets and higher-order multiples.

**Table 9:** Intrauterine insemination cycles in 2012

IUI	Cycles	Deliveries/ cycles	Gestational order		
			Singleton	Twin	≥Triplets
**Husband**	5,372	12.3%	89.4%	9.3%	1.4%
**Donor**	1,029	17.8%	88.0%	8.2%	3.8%

#### Cumulative and total delivery rate

The cumulative delivery rate corresponds to the number of deliveries resulting from one initiated or aspirated ART cycle including the cycle when fresh embryos are transferred, and subsequent frozen/thawed embryo transfers. This rate is used when less than the total numbers of embryos fresh and/or frozen/thawed have been utilised from one ART cycles. If all embryos are used, it is referred to as total delivery rate. Cumulative deliveries are calculated by adding deliveries derived from fresh plus frozen transfers. In future years, it will be possible to calculate cumulative events by each person. In 2012 the cumulative delivery rate in Latin America reached 28.1% (Table 10).

**Table 10:** Cumulative delivery rate in autologous IVF/ICSI cycles with at least one oocyte recovered in 2012.

	(n)	Delivery rate per OPU
**Total OPU**	29,824	
**Deliveries IVF/ICSI**	6,481	21.7%
**Deliveries FET**	1,912	6.4%
**Cumulative delivery**	8,393	28.1%

#### Fertility preservation

Ninety five centres from 10 different countries reported 1,764 initiated cycles for fertility preservation. Of these, 93 were carried out for cancer and 1,258 for social reasons, and in 413 cases the reason was not available. Overall, the mean age of women undergoing this procedure was 36 years (17 to 48 years). The mean number of oocytes preserved was 6 (from 0-39). In the most cases, the preferred technique for cryopreservation was vitrification, which represented 99% of the cycles. There was only one report of ovarian hyperstimulation syndrome and one report of haemorrhage.

#### Complications

Clinics reported 145 cases of moderate to severe ovarian hyperstimulation syndrome, corresponding to a rate of 0.5%. Other less frequent complications included 11 cases of haemorrhage and four cases of infection. It is likely, however, that complications are mis-registered.

#### Discussion

This is the 24^th^ version of the RLA, which has been published continuously since 1990. The RLA covers the vast majority of ART procedures carried out in Latin America. Over the years it has evolved, and now includes the collection and analysis of more complex information, allowing readers to download the registry in PDF file from our web page (http://www.redlara.com). 

With the 2010 register, an individualized case-by-case register was implmented, making it the first multinational registry to use this form of data entering. The software used was developed by RLA, and was field-tested in several institutions in the region. To implement this new software, workshops were carried out in different countries, and we believe, that the programme is still in a developmental phase, and continuous check-in systems are being incorporated as problems arise during its implementation.

One of the important requirements of any national or regional registry is to agree on common terminology. All clinics reporting to RLA use the glossary defined in 2009 by the International Committee for Monitoring Assisted Reproductive Technologies and the World Health Organization (Zegers-Hochschild *et al*., 2009). Another requirement for a reliable registry is to implement an external and independent accreditation programme, with the autonomy to check the quality and the validity of the data voluntarily reported by every participating institution. This has been successfully implemented by REDLARA since the year 2000.

In 2012, 155 centres of 14 countries reported 47,326 assisted reproduction technology cycles. Compared with 2011, this represents an increase of 12.9% in the number of cycles and 10 more centres (Zegers-Hochschild *et al*., 2013). In this year, the use of ICSI instead of conventional IVF continued to be the preferred insemination procedure. In 2012, ICSI represented 85% of oocyte pick-ups, which has remained almost unchanged since 2008.

The age of women undergoing IVF-ICSI cycles continues to increase. In 2012, the proportion of initiated IVF-ICSI cycles in women aged 35-39 years was 39%, similar to 2011 when it was 38% (Zegers-Hochschild *et al.*, 2013). In 2012, however, the proportion of IVF-ICSI cycles in women aged 40 years or over reached 31%, compared with only 17% in 2011. Furthermore, 10% of autologous IVF-ICSI were carried out in women aged 43 years or over. As the age of the female partner is one of the most important prognostic factors, this demographic reality is important to consider when analysing regional trends in number of embryos transferred and other markers of therapeutic outcomes.

The delivery rate per oocyte retrieval in autologous fresh IVF-ICSI reached 21.7% (21.4% in 2011), and the cumulative delivery rate reached 28.1% (27.5% in 2011). When examining delivery rate separately in IVF and ICSI, the higher rate in IVF must be considered with caution because of the lack of randomization of treatment alternatives.

It is worth mentioning that a delivery rate per embryo transfer of 10.25% (Figure 2a) with a spontaneous abortion rate of only 20.4% in women over 42 years seems too good, but in small numbers, this can be the result of mere chance. It is also possible that, in this age category, autologous IVF is only offered to a sub-group of women exhibiting optimum fertility markers.

The mean number of transferred embryos in autologous fresh IVF-ICSI decreased from 2.4 in 2010 to 2.2 in the actual report (Zegers-Hochschild *et al*., 2012). In most cases, two embryos were transferred. it is still concerning that, in one-quarter of embryo transferes, more than three embryos were transferred, and in 4%, four or more embryos were transferred.

Both in autologous IVF-ICSI and oocyte donation cycles, the transfer of more embryos resulted in a high proportion of triplets and higher order deliveries. Interestingly, the increase in the risk of twin-deliveries and triplet and higher order deliveries was only evident when three embryos were transferred.

As shown in this and previous reports, even twin deliveries increase the risk of preterm birth and perinatal mortality (Zegers-Hochschild *et al*., 2013). We believe that the main reason to transferring more embryos is the pressure from patients and clinicians to achieve pregnancy as early as possible, without considering the risk of multiple deliveries and associated prematurity. In Latin America, most patients undergoing infertility treatment are self-funding, and are not eligible for reimbursement by national or private health insurances.

This report, however, is quite reassuring, as the results associated with eSET and eDET, especially in younger patients undergoing IVF- ICSI, and oocyte delivery cycles, are quite acceptable. Therefore, clinicians and patients under 35 years should consider the transfer of one or at the most two embryos, and cryopreserve the rest for delayed transfer.

This report examines observational data, so the comparison of results cannot be considered hard evidence in favour or against certain procedures. For example, PGD was not associated with either a significant increase in the delivery rate, nor a reduction in the spontaneous abortion rate.

This might be explained by the fact that the number of procedures is still low and RLA does not register differently PGD and PGS. Furthermore, the selection of women having PGD can be very different to the rest of the population, even when stratified by age. The same applies for assisted hatching, which does not increase delivery rate, as no statistical significance was reached; however, caution must be exercised when analysing these data.

The frequency of complications associated with assisted reproduction technology procedures was rather low; only 145 cases of ovarian hyperstimulation synderome were reported, which represented a risk of 0.5% of initiated cycles. Furthermore, only 11 cases of genital haemorrhage and one case of infection were reported. Nevertheless, this low frequency might represent a recollection bias, which needs to be improved.

This is the sixth report of IUI cycles. Clinics reported 5372 IUI with husband’s semen, and 1029 cycles with donor semen. These figures are lower than those reported in 2011, or even lower than those reported in 2009, when 13,410 IUI-H and 2,430 IUI-D cycles were reported (Zegers-Hochschild *et al.*, 2011, Zegers-Hochschild *et al.*, 2013). This may be explained by the labour-consuming work that represented adapting IUI services into an individualized case-by-case register.

In summary, this is the third case-by-case register published by the RLA. It is reassuring for patients and clinics that the results of assisted reproduction technology procedures carried out in the region are similar or even better than many countries (Sullivan *et al.*, 2004; Ferraretti *et al*., 2013, Zegers-Hochschild *et al.*, 2013). Nevertheless, REDLARA has to enforce the reduction in the number of embryos transferred in IVF-ICSI and oocyte donation cycles, in order to prevent multiple births, or at least, high-order multiples and decrease the corresponding perinatal complications.

## Supplementary Data PARTICIPATING INSTITUTIONS

**ARGENTINA**
- Instituto de Fertilidad Asistida
- Centro de Estudios en Ginecología y Reproducción (CEGYR)
- Centro de Salud Reproductiva (CER)
- Centro de Estudios en Reproducción Humana (CERH)• Centro Integral de Ginecología, Obstetricia y Reproducción (CIGOR)
- Centro de Investigaciones en Medicina Reproductiva (CIMER)
- Centro de Medicina Reproductiva Bariloche
- Centro de Estudios en Reproducción y Procedimientos de Fertilización Asistida (CRECER)
- FECUNDITAS
- FERTILAB
- Centro de Reproducción FERTLEQUIP
- Centro especializado en tratamientos para la mujer (GENS)
- Hospital de Clínicas
- FECUNDART
- Instituto de Medicina Reproductiva
- Centro de Reproducción, Servicio de Ginecología, Hospital Italiano
- Mater, Medicina Reproductiva
- Nascentis, Medicina Reproductiva
- HALITUS, Instituto Médico
- Unidad de Fertilidad, Clinica de Prado
- PREGNA, Medicina Reproductiva
- PROCREARTE
- Fertilidad San Isidro
- SARESA, Salud Reproductiva Salta
- VITAE, Medicina Reproductiva

**BOLIVIA**
- CENALFES

**BRASIL**
- ANDROLAB, Clinica y Laboratorio de Reproducción Humana y Andrología
- ANDROFERT, Centro de Referencia en Reproducción Masculina
- FERTIVITRO, Centro de Reproducción Humana
- BIOS, Centro de Medicina Reproductiva
- Centro de Reproducción Humana de Campinas
- CEDILON, Laboratorio de Reproducción Humana
- Centro de Medicina de Reproducción LTDA
- Clinica de Fertilidad GERAR
- VIDA, Centro de Fertilidad REDE D’OR
- Clinica FERTWAY
- NASCER, Medicina Reproductiva Ltda.
- ORIGINARE, Centro de Investigación y Reproducción Humana
- CLINIFERT, Centro de Reproducción Humana
- CONCEPTUS, Centro de Reproducción Asistida de Cear
- CONCEBER, Centro de Medicina Reproductiva
- Clínica ORIGEN
- Centro de Reproducción Humana CONCEPTION
- Centro de Reproducción Humana MONTELEONE
- Fértile Diagnósticos
- CEERH, Centro especializado en Reproducción Humana
- EMBRYOLIFE, Instituto de Medicina Reproductiva
- Centro de Reproducción Humana, Endoscopia y Medicina Fetal de Bahía (CENAFERT)
- Instituto VERHUM
- Clínica FERTIBABY
- FECUNDA, Reproducción Humana
- FELICCITA, Instituto de Fertilidad Ltda.
- HUMANA, Medicina Reproductiva (Ex centro de Reproducción asistida FEMINA)
- FERTILITY, Centro de Fertilización Asistida de Campo Grande
- FERTILITY, Centro de Fertilización Asistida
- REPROFERTY
- FERTICLIN, Clínica de Fertilidad Humana
- GENESIS, Centro de Asistencia en Reproducción Humana
- FERTIPRAXIS, Centro de Reproducción Humana (Ex Fert. Gin. y Obst. de Barra)
- GERA, Grupo de endoscopia y Reproducción Asistida• Núcleo de Reproducción Humana Do Hospital Moinhos de Vento
- Instituto de Saude Da Mulher, Cegonha Medicina Reproductiva
- IVI Sao Paulo, Chedid Grieco S.A.
- PRIMORDIA, Medicina Reproductiva (Huntington RJ)
- Hospital de Clínicas de Riberao Preto
- HUNTINGTON, Centro de Medicina Reproductiva
- JULES WHITE, Centro de Medicina Reproductiva
- Instituto Brasileiro de Reproducción Asistida, IBRRA
- IMR, Instituto de Medicina Reproductiva e Fetal
- INSEMINE, Centro de Reproducción Humana • Servicio de Reproducción Humana Del Hospital y Maternidad Santa Johana
- FERTILITAT, Centro de Medicina Reproductiva
- Clínica MATRIX
- Centro de Reproducción Humana Nilo Frantz
- Clínica ORIGEN
- Clínica PRO-CRIAR, Medicina Reproductiva
- Clínica PRO NASCER
- REPRODUCTION, Clínica Urológica y Centro de Reproducción Humana Ltda.
- Centro de Reproducción Humana De San Jose de Rio Preto
- GENESIS, Centro de Reproducción Humana
- Centro de Reproducción Humana Prof. Franco Junior
- Centro de Ensino y Pesquisa en Reproducción Asistida (Centro de Rep. Asist. Hospital Da ASA SUL)
- LIFE, Reproducción Humana

**CHILE**
- UMR Clínica de la Mujer Antofagasta
- Centro de Estudios Reproductivos (CER)
- Unidad de Medicina Reproductiva, Clínica Alemana
- Unidad de Medicina Reproductiva, Clínica las Condes • Unidad de Medicina Reproductiva, Clínica de la Mujer
- Programa e Fertilización Asistida I.D.I.M.I.
- Clínica Monteblanco
- Centro de Fertilidad y Medicina Reproductiva Concepción S.A.

**COLOMBIA**
- Centro FECUNDAR, Cali
- Unidad de Fertilidad del COUNTRY LTDA.
- Asociados en Fertilidad y Reproducción Humana
- MEDIFERTIL
- Centro Médico IMBANACO
- Instituto de Fertilidad Humana S.A.S. (INSER)
- IN SER, Instituto Antioqueño de Reproducción
- PROCREAR
- PROFAMILIA-FERTIL
- Unidad de Fertilidad, Procreación Medicamente Asistida
- Unión Temporal IN SER EJE Cafetero

**ECUADOR**
- Clínica de Medicina Reproductiva BIOGEPA
- Centro Ecuatoriano de Reproducción Humana
- Clínica INFES
- Instituto Nacional de Investigación de Fertilidad y Esterilidad (INNAIFEST)
- CONCEBIR, Unidad de Fertilidad y Esterilidad
- Unidad de Fertilidad Hospital Alcívar

**GUATEMALA**
- Centro de Reproducción Humana S.A. (CER)

**MEXICO**
- Biología de la Reproducción Humana, Cirugía Reproductiva Gin. y Obst. (INSEMER) • Centro de Diagnóstico Ginecológico
- CEMAIN
- Clínica de Biología de la Reproducción
- Unidad de Reproducción Asistida del Servicio de Reproducción Humana del C.M.N. 20 de Noviembre • Instituto para el estudio de la Concepción Humana IECH
- Centro de Reproducción Asistida del Hospital Español (HISPAREP)
- Centro de Reproducción Asistida del Occidente
- Centro de Reproducción Asistida de Saltillo
- Centro Universitario de Medicina Reproductiva
- EMBRYOS POLANCO SA de CV
- Fertility Center Cancún
- Ginecología y Reproducción Asistida GYRA
- Unidad de Medicina Reproductiva del Hospital Ángeles del Pedregal
- Instituto Mexicano de Alta Tecnología Reproductiva S.C. (INMATER)
- Instituto de Medicina Reproductiva del Bajío (IMER), sede Guadalajara • Instituto IMER de Tijuana
- Instituto Médico de la mujer (RED CREA)
- Instituto de Ciencias en Reproducción Humana, sede Guadalajara
- Instituto de Ciencias en Reproducción Humana, sede Matamoros
- Centro especializado para la atención de la mujer
- INGENES
- Instituto Valenciano de Infertilidad (IVI) México• Instituto de Ciencias en Reproducción Humana (VIDA), sede León
- Médica Fértil, San Luis de Potosí
- Centro de Medicina Reproductiva FILIUS
- Centro especializado en esterilidad y Reproducción Humana

**NICARAGUA**
- Centro de Fertilidad de Nicaragua

**PANAMA**
- IVI Panamá S.A.

**PERU**
- Clínica CEFRA, Centro de Fertilidad y Reproducción Asistida
- Centro de Fertilidad y Ginecología del Sur (CFGS)
- FERTILAB, Laboratorio de Reproducción asistida
- Clínica Miraflores, Instituto de Ginecología y Fertilidad
- Grupo Pranor, Clínica CONCEBIR
- Grupo Pranor, Instituto de Ginecología y Reproducción

**REPUBLICA DOMINICANA**
- Instituto de Reproducción y Ginecología del CIBAO (IREGCI)
- PROFERT (Programa de Fertilización Asistida y Medicina Perinatal)

**URUGUAY**
- Centro de Esterilidad Montevideo (CEM)
- Centro de Reproducción Humana del Interior

**VENEZUELA**
- FERTILAB
- UNIFERTES
- EMBRIOS, Centro de Fertilidad y Reproducción Humana, Hospital de Clínicas de Caracas
- GENESIS, Unidad de Fertilidad y Reproducción
- UNISARE
- Instituto Venezolano de Fertilidad
- Laboratorios In Vitro de Venezuela
